# Perceptions of Patients Regarding Mobile Health Interventions for the Management of Chronic Obstructive Pulmonary Disease: Mixed Methods Study

**DOI:** 10.2196/17409

**Published:** 2020-07-23

**Authors:** Meshari F Alwashmi, Beverly Fitzpatrick, Jamie Farrell, John-Michael Gamble, Erin Davis, Hai Van Nguyen, Gerard Farrell, John Hawboldt

**Affiliations:** 1 Memorial University of Newfoundland and Labrador St. John’s, NL Canada; 2 School of Pharmacy, Faculty of Science University of Waterloo Waterloo, ON Canada

**Keywords:** mhealth, COPD, health technology, smartphone, mobile phone

## Abstract

**Background:**

Using a mobile health (mHealth) intervention consisting of a smartphone and compatible medical device has the potential to enhance chronic obstructive pulmonary disease (COPD) treatment outcomes while mitigating health care costs.

**Objective:**

This study aims to describe the demographics, use, and access to smartphones of patients with COPD. It also aims to explore and develop an understanding of potential facilitators and barriers that might influence patients using mHealth interventions for COPD management.

**Methods:**

This was an explanatory, sequential mixed methods study. Patients who attended respirology clinics completed a questionnaire on technology access and use. We conducted semistructured individual interviews with the patients. Interview topics included the following: demographics, mHealth use, perceptions toward challenges of mHealth adoption, factors facilitating mHealth adoption, and preferences regarding features of mHealth interventions for COPD management.

**Results:**

A total of 100 adults completed the survey but 22 participants were excluded because they were not diagnosed with COPD. Of these, 10 patients with COPD participated in the interview. The quantitative component revealed that many patients with COPD owned a mobile phone, but only about one-fourth of the participants (18/77, 23%) owned a smartphone. The likelihood of owning a smartphone was not associated with age, sex, marital status, or geographical location, but patients with high educational status were more likely to own a smartphone. The qualitative component found that patients with COPD, in general, had a positive attitude toward mHealth adoption for COPD management, but several facilitators and barriers were identified. The main facilitators of mHealth adoption are possible health benefits for patients, ease of use, educating patients, and credibility. Alternatively, the barriers to adoption are technical issues, lack of awareness, potential limited uptake from older adults, privacy and confidentiality issues, finances, and lack of interest in mHealth

**Conclusions:**

It is important to understand the perceptions of patients with COPD regarding the adoption of innovative mHealth interventions for COPD management. This study identifies some potential facilitators and barriers that may inform the successful development and implementation of mHealth interventions for COPD management.

## Introduction

Although chronic obstructive pulmonary disease (COPD) is a preventable and treatable disease, it is currently the third leading cause of death worldwide [1,2]. According to the Canadian Institute for Health Information, COPD now accounts for the highest rate of hospital admission and readmission among major chronic illnesses in Canada [3]. The Conference Board of Canada has stated that the combined direct and indirect costs of COPD will increase from just under Can $4 billion (US $2.93 billion) in 2010 to Can $9.5 billion (US $6.96 billion) by 2030, an increase of 140% [4]. These costs include direct costs (including drugs, hospitals, and physicians) and indirect costs (including long-term disability losses and mortality). Advances in technology have the potential to enhance both pharmacological and nonpharmacological interventions for COPD management.

This surge in computing power and mobile connectivity has led to the inception of mobile health (mHealth), which can transform clinical research and health care [5]. mHealth is defined by the National Institutes of Health as the “use of mobile and wireless devices to improve health outcomes, healthcare services, and health research.” An mHealth intervention could also include the use of a medical device that is compatible with a smartphone. Previous research suggests that mHealth interventions may benefit patients with many chronic health conditions, including COPD [6]. Some of these benefits include improving the knowledge about COPD, increasing physical activity, and reducing exacerbations [7-9]. Alwashmi et al [6] noted that the current literature on the role of smartphones in reducing COPD exacerbations is limited, but they suggest that smartphone interventions may reduce COPD exacerbations. To potentially enhance the adoption and outcome of mHealth interventions, key users should be involved in the development of these interventions.

The International Organization for Standardization (ISO) 9241-210 defines human-centered design (HCD) as “an approach to systems design and development that aims to make interactive systems more usable by focusing on the use of the system and applying human factors/ergonomics and usability knowledge and techniques” [10]. The ISO uses the term HCD instead of user-centered design to “address impacts on a number of stakeholders, not just those typically considered as users” [10]. However, in practice, these terms are often used synonymously.

Many researchers use mHealth to assist in the management of chronic diseases; nevertheless, gaps still exist regarding the development process of these mHealth interventions [11]. Testing mHealth interventions with patients has revealed preferences and concerns unique to the tested population [12-14]. Developing a COPD mHealth intervention with insights from patients will potentially improve the process and outcome of mHealth interventions. 

Patient perspectives toward using mHealth for COPD management are relatively unexplored [11,15]. A recent meta-analysis on the remote monitoring of patients with COPD concluded that some interventions may prove to be promising in changing clinical outcomes in the future, but there are still large gaps in the evidence base [16]. Noah et al [16] suggested that adding a qualitative component would give researchers insight into which elements best engage and motivate patients and health care professionals (HCPs).

To improve the success of mHealth interventions that target patients with COPD, we included patients in the development process. The lessons learned will bridge the knowledge gap between barriers and facilitators for mHealth uptake in COPD management. Key lessons learned will be offered as a guide for research and technology developers who are developing mHealth interventions for COPD management [12,14,17]. Furthermore, before implementing mHealth interventions, it is important to understand the use of and access to mHealth.

## Methods

### Purpose

This study aimed to describe the demographics, use, and access to smartphones of patients with COPD. It also aimed to assess whether demographic factors predict engagement with mHealth and to explore and develop an understanding of potential facilitators and barriers that might influence patients using mHealth interventions for COPD management.

### Study Design

First, participants completed a questionnaire about technology use and access. The findings from the questionnaire were used to develop the sample and questions for the qualitative phase [18]. A subset of participants were interviewed to provide deeper insights into technology access and use among patients with COPD. In this study, the quantitative component was fully completed before the design and implementation of the qualitative component. The results of the quantitative component were used to define the qualitative sample.

### Ethical Considerations

Ethical approval for this study was obtained from the Newfoundland and Labrador Health Research Ethics Authority (HREB-2017-194). Before agreeing to participate, all subjects were informed about the nature of the research project, possible risks and benefits, and their rights as research subjects. All participants in the interview completed a written consent form and were given a copy.

### Quantitative Phase

The quantitative phase aimed to describe the demographics, use, and access to smartphones among patients with COPD. It also aimed to assess whether demographic factors predicted engagement with mHealth. The results were used to define the qualitative sample and create the questions required for the qualitative phase.

#### Recruitment and Study Setting

Participants were recruited during routine visits to their respirologists at outpatient respirology clinics in St John’s, Newfoundland and Labrador, Canada. There are 3 clinics in the city. Participants were eligible for the study if they met the following inclusion criteria:

1. A COPD diagnosis (self-report).
2. Aged ≥30 years at study enrollment.
3. Ability to answer questionnaires in English.
4. Ability to provide informed consent.

Patients who attended respirology clinics received a consent cover letter for research. The cover letter included a questionnaire about technology access and use (Multimedia Appendix 1). After reading the cover letter, interested participants completed the questionnaire and submitted it in a locked box (red box) available at the respirology clinic.

The last section of the questionnaire included a question about the participant’s interest in participating in an interview regarding the same topic. Interested participants provided their contact information and placed it in another box (blue box) located at the respirology clinic.

We separated the contact information from the questionnaire to ensure that the questionnaire was kept anonymous. Each questionnaire had a unique identifier. Only the primary investigator was able to link the questionnaire with the contact information. The sample size (n=77) is comparable to that reported by Granger et al [19] and Kayyali et al [20] who assessed the use of technology among patients. Patients did not receive remuneration for completing the questionnaire.

#### Data Collection

The questionnaire was adapted from Ramirez et al [21] to assess how patients use various types of mobile technology and to what extent they use mHealth. In addition, the questionnaire was used to assess the need for and interest in using mobile health technology, such as mobile phone apps and social media, to help manage health. Similar to Ramirez et al [21] the questionnaire was not validated due to the lack of standardized instruments regarding the use and access to mHealth; however, the questions were consistent with previously published literature in the field.

The questionnaire also included demographic information such as age, sex, race, ethnicity, primary language spoken, annual household income, and education level. In addition, participants were asked about their mobile phone ownership and if their mobile phone had internet capabilities. They were also asked if they used the internet on their mobile phones to learn about their health. Afterward, the participants were asked about their knowledge of mobile phone apps, if they used such apps, and if they were currently using any mobile health apps. In addition, participants identified individuals who they might rely on to use mobile phones and/or mobile apps for them (eg, partner, child, friend).

#### Statistical Analysis

A database of the questionnaire results was created using unique nonidentifying numbers. The information was password-protected. Before conducting the analysis, data were cleaned, coded, and entered into SPSS version 25.0 (IBM Corporation). Unclear or incomplete survey items were flagged for queries. These were brought to the attention of the research team, then each item was discussed, and a decision concerning its eligibility and entry was made.

Baseline characteristics of participants were summarized with percentages for categorical variables and means and standard deviations for continuous variables. Crude and adjusted odds ratios were measured using univariate and multivariable logistic regression analyses to determine if smartphone ownership was independently associated with age (30-64 years/65 years or older), sex (female/male), marital status (in a relationship/not in a relationship), education level (less than high school/high school/more than high school), and geographical location (rural area/small population center/medium or large population center). All these variables have a known association with smartphone ownership and have been previously reported in the literature [22]. All statistical tests were performed with an alpha significance level of .05.

### Qualitative Phase

We used a descriptive qualitative research design grounded in pragmatism [23,24]. Using a qualitative methodology allowed us to achieve an in-depth, contextualized picture of how a diverse sample of patients with COPD think and feel about the possibilities and challenges of using mHealth. This has pragmatic value, as mHealth is an emerging option for delivering health care.

#### Recruitment and Study Setting

Once all the questionnaires were collected and analyzed, we only contacted participants who agreed to participate in the interview. On the bases of the demographic information collected from the questionnaire, a purposeful sampling strategy was used to identify key informants who could provide rich and diverse interview data. We also used a criterion-based selection [24] so that we could categorize participant characteristics such as age, familiarity with mHealth, smartphone ownership, and years living with COPD.

After interviewing 7 to 8 patients, we reached saturation, as we were not gathering new information. However, we continued interviewing until 10 patients were interviewed to strengthen the validity of inferences [25]. Our final sample size was comparable with similar qualitative studies [15,26].

The study was conducted in St John’s, Newfoundland and Labrador, Canada. We conducted interviews at the Memorial University of Newfoundland and others at the participants’ homes. Participants were recruited from April to August 2018. After completing the interviews, participants were offered a gift card (Can $30 [US $1.3]).

#### Data Collection

We conducted individual semistructured interviews to gain an understanding of the lived experiences of patients with COPD in relation to using mHealth [27,28]. Using semistructured interviews allowed the interviewer to begin with a broad question to direct the focus of the interview and then to provide an opportunity for patients to bring forth their thoughts and feelings about the phenomena they thought were important [27,28]. The interview prompts are available in Multimedia Appendix 2. If participants identified that they had not used mHealth, they were asked questions pertaining to why they had not used mHealth (barriers). However, we did not ask them about facilitators because we did not think they had the experience needed to answer these questions. To facilitate discussions, the interviews were conversational in nature, and items were not asked verbatim or in the order presented. As the study progressed, emerging issues were explored with subsequent participants to refine the themes.

The interview questions and prompts were informed by findings from the literature and input from the authors, who have diverse backgrounds including in mHealth, pharmacy, nursing, medicine, respirology, family medicine, education, and qualitative research. They were also informed by interviewing HCPs regarding the use of mHealth in COPD management [29]. Finally, the interview questions were informed by the results of the quantitative phase.

The interviews were recorded to enable transparent and accurate transcriptions. Interview lengths ranged from 20 to 40 min. Topics included demographics, mHealth usage, perceptions toward challenges of mHealth adoption, factors facilitating mHealth adoption, and preferences regarding features of the mHealth intervention for COPD management. Owing to the large amount of data, preferences regarding features of the mHealth intervention will be published in another article. Data consisted of about 4 hours of interview time with approximately 100 pages of transcription.

#### Data Analysis

The interviews were transcribed verbatim and compared against the digital recordings to ensure the accuracy of the content. Identifying information (names) was removed to ensure anonymity. We used NVivo (version 12; QSR International) to organize the data and examine the words, including frequency counts, as in classical content analysis [30]. All data were analyzed, but we only coded the data that were relevant for answering the research questions, as recommended by Saldana [31], Wolcott [32], and Yin [33]. An audit trail was created to keep track of all analytic decisions [34].

After using NVivo, we used first cycle coding that was both structural and holistic [31], meaning that we used the interview prompts and the literature to guide some of the coding. One researcher analyzed the transcripts and developed a set of themes and subthemes and then obtained input from a second researcher. In the second cycle of coding, the 2 researchers independently coded the data using pattern coding to develop themes [31]. They then discussed commonalities and differences in their coding and theme development until consensus was reached. Qualitative data analysis is mainly inductive as the researcher does not approach the data with a pre-existing hypothesis that is meant to be supported or refuted. Instead, the researcher reads and rereads the data, using specific information to generate more general ideas that may take the form of themes or explanations. For this study, we had already analyzed data from health care providers [29]. We used a deductive approach to look for patient data that would either support or disconfirm the HCP results from the point of view of the patients. In addition, inductive analysis was also used to search for new ideas not mentioned by the HCPs. The iterative process continued as these analyses were conducted to find commonalities, differences, and new patterns in thinking.

### Mixed Methods Integration

In addition to the integration at the study design level, we implemented integration at the methods, interpretation, and reporting levels [35]. We implemented integration at the methods level in 2 ways: connecting and building. Connecting occurs when a researcher links one type of data to another type of data through sampling [35]. In this study, we used data from the questionnaire to purposefully sample participants for follow-up interviews. Specifically, we were able to categorize participant characteristics such as age, familiarity with mHealth, smartphone ownership, and years living with COPD. Obtaining information from a diverse group may generate a more complete picture, reveal patterns that would otherwise go unnoticed, and may also help identify novel relationships between variables and concepts [18]. Building occurs when one database informs the data collection approach of the other [35]. In this study, we used the questionnaire responses to develop some aspects of the interview guide. This allowed us to gain further insights regarding mHealth use. For example, knowing if patients used mHealth in the past was helpful in understanding the facilitators of mHealth adoption.

Furthermore, we implemented integration at the interpretation and reporting levels. We used both integrations through narratives and the use of a joint display [35,36]. Integration through a narrative occurs when a researcher describes the quantitative and qualitative findings in a single report or series of reports [35]. In this paper, we described the findings in a single report and used the contiguous approach, in which qualitative and quantitative findings are reported in different sections. Finally, we used a joint display to provide a structure to discuss the integrated analysis [36].

## Results

### A Cross-Sectional Survey of Patients

#### Demographics

A total of 100 adults completed the survey from January to November 2018. Only 77 participants reported that they were diagnosed with COPD and were included in the analysis. Table 1 provides an overview of the demographic and health information of the sample. Table 1 indicates that most participants were aged 55 years or older, and 61.5% had an annual income of less than $40,000. About 70% had earned at least a high school diploma, and there was a mixture of rural and urban participants. Participants had a range of comorbidities, and almost 65% were taking 5 or more medications.

**Table 1:** Participant demographics and health information (N=77).

Variables		Values, n (%)	
**Age (years; n=77)**			
	30-34		2 (2.6)
	45-54		3 (3.9)
	55-64		15 (19.5)
	65 or older		57 (74)
**Sex (n=74)**			
	Female		44 (59.5)
	Male		30 (40.5)
**Marital status (n=74)**			
	Married		44 (59.5)
	Common law		6 (8.1)
	Single (never married)		6 (8.1)
	Widowed, separated, or divorced		18 (24.3)
**Income (Can $; n=52)**			
	Under 20,000		14 (26.9)
	20,000-39,000		18 (34.6)
	40,000-59,000		6 (11.5)
	60,000-79,000		4 (7.7)
	80,000-150,000		8 (15.4)
	Over 150,000		2 (3.8)
**Employment (n=68)**			
	Employed full time		6 (8.8)
	Employed part time		2 (2.9)
	Self-employed		2 (2.9)
	Retired		52 (76.5)
	Unemployed		6 (8.8)
**Education level (n=68)**			
	Less than high school		14 (20.6)
	High school equivalency (GED)		9 (13.2)
	High school		25 (36.8)
	College/trade		10 (14.7)
	Bachelor’s degree		5 (7.4)
	Master’s degree		4 (5.9)
	PhD/MD/JD		1 (1.5)
**Population size (n=71)**			
	Rural area (with a population less than 1000)		18 (25.4)
	Small population center (with a population between 1000 and 29,999)		23 (32.4)
	Medium population center (with a population between 30,000 and 99,999)		6 (8.5)
	Large urban population center (with a population of 100,000 or more)		24 (33.8)
**Self-reported comorbidities^a^ (n=65)**			
	Cancer		18 (28.1)
	Diabetes		15 (23.4)
	Heart disease		14 (21.9)
	Skeletal or muscular disease		12 (18.8)
	Kidney disease		4 (6.3)
	Mental health issues		2 (3.1)
**Medication intake (n=68)**			
	None		2 (2.9)
	1-2		10 (14.7)
	3-4		12 (17.6)
	4-6		16 (23.5)
	More than 6		28 (41.2)

^a^Some patients reported several comorbidities.

#### mHealth Technology Ownership

Table 2 illustrates the findings regarding mHealth technology ownership. A total of 73% (56/77) participants owned a mobile phone, but only about one-fourth of the participants, 23% (18/77), owned a smartphone. The number of iPad or tablet owners was slightly higher than that of smartphones at 33% (25/77). In terms of the availability of a smartphone in the household, 27% (21/77) participants stated that a person in the household owns a smartphone. Although only 23% (18/77) participants reported having a smartphone, 29% (22/77) participants were able to access the internet via their phone, suggesting a higher number of smartphones than initially reported. About a third of participants 35% (27/77) owned a blood pressure monitor, and 22% (17/77) participants owned a glucometer.

Logistic regression was performed to ascertain the association of age, sex, marital status, education level, and geographical location with the likelihood that participants owned a smartphone. Owing to missing observations, the true sample used in the regression was 65 of 77.

We measured crude odds ratios to determine whether smartphone ownership was independently associated with the occurrence of predictor variables. The likelihood of owning a smartphone was reduced in participants earning less than a high school diploma (crude odds ratio [cOR] 0.11, 95% CI 0.02-0.64; *P*=.01) and participants earning a high school diploma (cOR 0.14, 95% CI 0.04-0.5; *P*=.002) compared with participants who received education beyond high school.

The logistic regression model was statistically significant, χ^2^_7_=15.8, *P*=.01. The model explained 30.4% (Nagelkerke *R*^2^) of the variance in smartphone ownership and correctly classified 77% of cases. Sensitivity was 50%, and specificity was 89%. Of the 5 predictor variables, only 2 were statistically significant, and both were related to education level (as shown in Table 3). The likelihood of owning a smartphone was reduced in participants earning less than a high school diploma (adjusted odds ratio [aOR] 0.12, 95% CI 0.017-0.86; *P*=.03) and participants earning a high school diploma (aOR 0.13, 95% CI 0.03-0.54; *P*=.005) compared with participants who received education beyond high school.

**Table 2:** Mobile health technology ownership (N=77).

Mobile health technology ownership	Values, n (%)
Mobile phone	56 (72.7)
Smartphone	18 (23.4)
iPad	25 (32.5)
Availability of a smartphone in the household	21 (27.3)
Internet access through a mobile phone	22 (28.6)
Spirometer/peak flow meter	4 (5.2)
Glucometer	17 (22.0)
Blood pressure monitor	27 (35.1)
Heart rate monitor	10 (13.0)
Accelerometer/activity counter	3 (3.9)
Scale	15 (19.5)
Thermometer	18 (23.4)

**Table 3:** Logistic regression predicting the likelihood of smartphone ownership.

Variables		Adjusted odds ratio (95% CI)		*P* value		Crude odds ratio (95% CI)		*P* value	
**Age (years)**									
	30-64		2.24 (0.57-8.77)		.25		1.97 (0.65-6.04)		.23
	65 or older		Reference		N/A^a^		Reference		N/A
**Sex**									
	Female		2.10 (0.54-8.19)		.29		1.03 (0.36-2.94)		.95
	Male		Reference		N/A		Reference		N/A
**Marital status**									
	In a relationship^b^		2.36 (0.494-11.29)		.28		1.63 (0.51-5.17)		.41
	Not in a relationship^c^		Reference		N/A		Reference		N/A
**Education level**									
	Less than high school		0.12 (0.02-0.86)		*.03* ^d^		0.11 (0.02-0.64)		*.01* ^c^
	High school^e^		0.13 (0.03-0.54)		*.005* ^d^		0.14 (0.04-0.50)		*.002* ^c^
	More than high school		Reference		N/A		Reference		N/A
**Population size**									
	Rural area		0.50 (0.09-2.76)		.43		0.35 (0.08-1.47)		.15
	Small population center		0.46 (0.11-1.92)		.29		0.61 (0.19-2.01)		.42
	A medium population center or a large population center		Reference		N/A		Reference		N/A

^a^N/A: not applicable.

^b^In a relationship includes being married or in common law.

^c^Not in a relationship includes being single, widowed, separated, or divorced.

^d^Significance level <.05.

^e^High school includes General Educational Development.

#### mHealth Technology Use

Table 4 highlights the findings related to mHealth technology use. Only a third of the participants 29% (20/77) understood the term “app.” Of these, 50% (10/20) used apps. Among app users, only 3/10 participants used health apps, and 7/10 participants were interested in using a health app. Six app users said they would be comfortable allowing their family members to access their health information, and 7 said they would be comfortable with their HCP accessing their health care information. The most common social media platform used was Facebook 38% (29/77). Among users of social media, 45% (13/29) reported using social media at least once a day.

Participants completed questions about their concerns regarding mHealth adoption. The first question was about concerns regarding smartphones. The following 3 options were chosen by participants: cost of smartphones 24.5% (21/77), reducing face-to-face interactions 20.4% (10/77), and not easy to use 18.4% (9/77). Of the participants who used apps (n=10), the following concerns about app use were chosen: worried about personal information disclosure (n=6), extra fees to use the app (n=3), apps use a lot of data (n=3), apps are not easy to use (n=1), and I do not know if they are effective (n=1). No participants chose “taking too much time to use” or “not recommended by a health care provider” as a concern arising from using apps.

**Table 4:** Mobile health technology use.

Variables			Values, n (%)
Understood the term “app” (n=77)^a^			20 (26)
Use apps (n=20)^b^			10 (50)
Use health apps (n=10)^c^			3 (30)
Interested in using health apps (n=10)			7 (70)
Comfortable allowing a family member to access health information (n=10)			6 (60)
Comfortable allowing a health care provider to access health information (n=10)			7 (70)
**Social media use (n=77)**			
	Facebook	29 (38)	
	Twitter	2 (3)	
	Instagram	2 (3)	
	Snapchat	1 (1)	
Interested in using social media to share health experience (n=77)			9 (12)
**Frequency of social media use (n=29)^d^**			
	Never	1 (3.4)	
	A few times a month	5 (17.2)	
	A few times a week	3 (10.3)	
	About once a day	7 (24.1)	
	More than once a day	13 (44.8)	

^a^Total study population.

^b^Sample population that understood the term “app.”

^c^Sample population that uses apps.

^d^Sample population that uses social media.

### Semistructured Interviews With Patients

We developed themes under 2 categories: facilitators and barriers that would influence the feasibility and use of mHealth. We have included details and examples to illustrate patients’ thoughts and beliefs. These findings expand on the barriers and facilitators reported previously by health care providers who treat patients with COPD [29]. Textbox 1 highlights the facilitators and barriers reported by patients.

**The facilitators and barriers to mobile health adoption.**

**Facilitators**

1. There are possible health benefits for patients
2. The software needs to be easy to use
3. Patients need to be educated on the use of mobile health (mHealth)
4. The credibility of mHealth needs to be evident

**Barriers**

1. There are technical issues with mHealth
2. Lack of awareness is a challenge
3. There may be limited uptake from the elderly
4. There are possible financial barriers
5. There may be privacy and confidentiality concerns
6. There was little interest in using an mHealth intervention

#### Demographics

A diverse group of 10 patients with COPD participated in face-to-face interviews. The mean age was 67.6 (SD 7.58) years, and the range was from 51 to 80 years. There were 4 females and 6 males. Participants stated that the mean number of years living with COPD was 8.4 (SD 4.45), and the range was from 3 to 15 years.

Most patients expressed interest in using mHealth to assist in the management of their COPD. In terms of smartphone ownership, 6 participants owned a smartphone, 2 owned a mobile phone, and 2 did not have either. Participants used their phones for different purposes, including communication, managing finances, gaming, and browsing the internet. One patient stated “I use it for everything. I never thought I’d see the day where I was dependant on my phone.” On the other hand, another participant stated that she did not use her smartphone beyond making phone calls: “I have a phone, but I am not smart enough to use it.” Some participants used it to monitor their physical activity: “I have a health thing on it and I look at it every once in a while just to see how many steps I’ve done that day because it...Improve health activity.” One patient used his smartphone to “get pollen reports and anything that will trigger a COPD attack...to do research on nutraceutical products or on COPD-related matters.” Four participants were enrolled in an mHealth intervention to manage their COPD.

#### Facilitators

Patients reported 4 facilitators, which are discussed below.

##### 1. There Are Possible Health Benefits for Patients

Participants agreed that mHealth has the potential to provide health benefits to patients. One patient who used a fitness tracker remarked:

I think it’s better for me to track what I do every day. It is going to make me feel better...That would help me a lot with my pains...I know how my day was and I know how I feel like.

Another patient who kept track of his vitals, weight, and medication intake stated that:

if I had been monitored, I might not have this broken arm...That should’ve been picked up on. I mean, I got the records there and you look back on it, I can easily look back on them now and say you had water retention, your resting heart rate was way too high, and your blood pressure was low. There’s something wrong.

Two patients described that mHealth could provide a sense of security and reduce hospitalization:

...it felt good to have that, you know, that security there at least that in those four months that I had it. So there was no guessing because you don’t know if you should or you shouldn’t go to a hospital.

##### 2. The Software Needs to Be Easy to Use

Usability was highlighted by many patients as an important factor in increasing the uptake of mHealth. One patient cautioned that he might not use an intervention if it was not easy to use:

I’m not getting into something that’s going to fill up my day ferreting around. But if it’s something that I’ve got to look at for five or ten minutes, I’m okay with that.

Participants who were enrolled in an mHealth intervention mentioned that there was “no trouble setting up. It’s all there, so all you had to do is turn it on.”

##### 3. Patients Need to Be Educated on the Use of mHealth

Patients learned how to use mHealth interventions via different sources. The majority asked their family members for assistance “...I’ll go to my 14-year-old who is generationally more apt to be able to teach me a new technology.” This was also mentioned by another patient who used a fitness tracker: “My nieces. They buy it for me and they set it up for me and everything.” Other patients taught themselves about mHealth interventions: “If I can read it, I can learn it...I generally research it myself” or used the library: “Also the libraries here will help you in any programs.” One patient said that her “...own care worker helps with it, I don’t know how to do it.” It was also recommended by some that technical support staff be available as a resource for patients to call when they needed technical help.

##### 4. The Credibility of mHealth Needs to be Evident

Some patients thought that the credibility of mHealth needs to be made evident. Some patients stated that they would use an mHealth intervention if it was recommended by their HCP: “if he told me that, I probably would try to do it.” This was reiterated by a patient who regularly monitored his COPD: “Doctor xx was the one that said to me...If you’re going to cope with this and keep it under control, you’re going to have to learn how to look after yourself.”

#### Barriers

Patients reported 6 barriers to mHealth adoption, which are discussed below.

##### 1. There Are Technical Issues With mHealth

A few patients expressed that they did not have the technical expertise to use mHealth. One patient expressed his concern: “I wouldn’t know how to turn a computer on. I’m not very good... You know, I never grew up with computers, but I have seen on.” This was reiterated by another patient: “It just looked way too complicated to download the app so I didn’t because I’m technology averse.” For patients who used mHealth, technical issues included limited cellular and Wi-Fi connections: “we travel out to the cabin every weekend and the cabin’s out in central Newfoundland out in Terra Nova and now it’s getting better now because these phone services getting better out there.” Another issue was moving and setting up the mHealth intervention components, such as a blood pressure monitor or a scale, when traveling: “...it’s not a problem. But when you go on vacation, sometimes you got to take this along with you and set it up somewhere.”

##### 2. Lack of Awareness is a Challenge

Many patients indicated that a lack of awareness is a barrier to mHealth adoption. One patient expressed this concern: “I’m not aware of everything that’s out there, but people need to be more aware of their COPD and know more about it.” In addition, a family member, who accompanied her mother in the interview, stated the following about her mother who has COPD:

I think it’s more she probably don’t know what her phone can do, right. But if you say to her okay we’re going to start using this, this is going to be useful and it’s going to be beneficial I’m sure she’d be game for it.

However, patients who participated in an mHealth intervention stated that their HCP recommended mHealth interventions when they were in the hospital or attended a community health event. Another patient mentioned that “it was advertised and I called in about it and they got in contact with me, set me up with it.”

##### 3. There May Be Limited Uptake by the Elderly

A few patients mentioned that they face issues in adopting technology because of their age. One patient stated, “I’m not generationally born into technology that is prevalent and considered a norm of the day.” This was also mentioned by another: “I try but I’m a little bit nervous, sometimes I’ll ask my daughter or someone else around because we didn’t grow up with the phones as you do today.”

##### 4. There Are Possible Financial Barriers

A few patients said they cannot afford the costs associated with mHealth, such as the cost of a smartphone. One patient expressed it this way: “there’s no way I’ll pay that money.” Expenses incurred through the use of data were also discussed: “it’s a bit expensive for like I’ve got no data right now because it’s all extra.” In addition to individual patient costs, one patient raised the concern of additional costs required by HCPs: “doctors are not going to do that without a fee.” In addition, some mHealth programs are limited to a certain period, which may increase costs.

##### 5. There May Be Privacy and Confidentiality Concerns

One patient thought privacy and confidentiality could be a barrier to mHealth adoption. She was open to sharing the results with an HCP but not to a family member: “I don’t want to make them worry (her family) because I told them nothing about my cancer... I don’t like to worry my family.” The rest of the patients were willing to share the results with their family members and HCPs: “I don’t care who sees it...They can put it in the Evening Telegram, doesn’t bother me.” This was echoed by another patient: “they (his family) watch over my shoulder like nothing else now.”

##### 6. There Was Little Interest in Using an mHealth Intervention

Some patients indicated that a lack of interest in mHealth is a major barrier to its adoption. One patient expressed this concern: “I do try to help myself but when it comes to using the phone and that stuff and the computer, it’s not for me.” This was also mentioned by another patient with limited technology experience: “I know on smartphones you can dial, you can play a game and some they can even watch movies probably, but I got no interest.” Another patient mentioned that they are too busy to include an mHealth intervention in their routine: “So my day is pretty filled with different things. So remembering is a problem. Sometimes I just say: To hell with it and I am not going to do it. But remembering is probably the biggest thing.”

When patients use mHealth, they may share the results with their HCP. This occurs if the mHealth intervention is not integrated in the health care system. One patient posited that some HCPs are not interested in reviewing the records brought by patients: “And my sheet, he didn’t even look at it...That’s, that’s depressing.”

## Discussion

### Principal Findings

We conducted an explanatory, sequential mixed methods study with patients and identified their perceptions regarding the use of mHealth for COPD management. The quantitative component revealed that over 70% of patients owned a mobile phone, but only about a quarter of the participants (18/77, 23.4%) owned a smartphone. The likelihood of owning a smartphone was not associated with age, sex, marital status, or geographical location. However, patients with a high educational status were more likely to own a smartphone. The qualitative component found that patients, in general, had a positive attitude toward mHealth adoption for COPD management, but several facilitators and barriers were identified. It is important to promote facilitators and address the barriers to optimize the successful implementation of mHealth interventions.

Using a mixed methods approach allowed us to produce a diverse sample of patients with COPD. The quantitative and qualitative components complemented each other to improve the validity of inferences and expand on why participants answered quantitative questions in a certain way. For example, the number of participants who stated that they had a smartphone (n=18) was lower than the number of participants who accessed the internet through their mobile phone (n=22), suggesting a lack of understanding of what a smartphone is. This was further explored during the interviews. Although some participants owned a smartphone, their use was limited to making phone calls and taking pictures. On the other hand, some participants did not own a smartphone, but they were able to enroll in an mHealth intervention and complete the program. As explained by one patient, “Eastern Health sets you up with everything. It’s so different, there’s nothing to it, it’s just hit the button, use your device and it’s so easy to use.” This finding highlights the need for education and confidence building among patients with COPD.

We created a joint display (Table 5) to clarify some of the barriers that were reported quantitatively. The table is organized by survey items. It merges the related quantitative results and provides typical comments from patients.

**Table 5:** Joint display of barriers to mobile health adoption.

Quantitative results: variables		Values, n (%)	Qualitative results: exemplar quotes	Interpretation of mixed methods findings
**Concerns regarding smartphones (N=77^a^)**				
	Cost of smartphones	21 (27.3)	“I can’t afford one (smartphone)” “I’ve got no data right now because it’s all extra” “we can always get one (smartphone)”	Costs include the cost of a smartphone and the data to enable its functionalities. However, some patients could afford to get a smartphone, or it could be provided by the health care system
	Not easy to use	9 (11.7)	“I wouldn’t know how to turn a computer on. I’m not very good...” “There’s nothing to it, it’s just hit the button, use your device and it’s so easy to use”	Although some participants owned a smartphone, their use was limited to making phone calls and taking pictures. On the other hand, some participants did not own a smartphone, but they were able to enroll in a mobile health intervention and complete the program. This finding highlights the need for education and confidence building among patients with COPD^b^
**Concerns regarding app use (N=10^c^)**				
	Worried about personal information disclosure	6 (60)	“I don’t want to make them worry because I told them nothing about my cancer...I just told my sister a week before I had my surgery...I don’t like to worry my family” “I don’t care who sees it...They can put it in the Evening Telegram, doesn’t bother me”	There was inconclusive evidence regarding confidentiality. Patients should have a choice in what to share and who should have access to their health information

^a^The total study population.

^b^COPD: chronic obstructive pulmonary disease.

^c^The sample population that uses apps.

### Comparison With Prior Work

Our study provides a meaningful contribution to the literature, as few prior studies have specifically examined the use of mHealth among patients with COPD. It is important to note that the published literature on mHealth access and use was focused on general and largely healthy populations, with little attention to individuals with chronic illnesses, such as COPD [37].

A study investigated smartphone ownership among the general public and reported a high smartphone adoption rate of 76% [20]. Ramirez et al investigated smartphone ownership in primary care clinics and found a high adoption rate of 76%; however, 96% of the participants were younger than 65 years. Among patients with COPD from several locations in the United States, we found lower smartphone adoption rates (47%), which was double the rate reported in our sample [38]. The qualitative data expand on this finding by clarifying that some smartphone owners use their device in ways similar to mobile phone owners. Knowing how to use the features of a smartphone, such as using apps, is necessary for using the mHealth intervention. This finding highlights the need for education and confidence building among some smartphone users to help them use their devices for COPD management.

Our logistic regression results support the claim that a lower level of education is associated with limited access to mobile devices [39-41]. Other researchers also found that the individuals more likely to use health apps tended to be younger and have higher incomes [38-41]. Our findings echo concerns about the relationship between mHealth access and health disparities [38]. Limiting mHealth interventions to smartphone or tablet owners could disproportionately benefit highly educated and wealthy individuals.

We also investigated the association between technology use and geographical location. Although we did not find differences between smartphone ownership among urban and rural patients with COPD, a report suggests that individuals living in rural areas are less likely to have smartphones than individuals not living in rural areas [42]. Previous studies suggest that among patients with COPD, living in rural areas was associated with a worse health status [43,44]. The authors suggest that the higher prevalence of COPD in rural areas could be linked to an increased proportion of older residents, a shortage of HCPs, the underuse of spirometry and pulmonary rehabilitation, and problems with access to medical care [43,44]. Limited access to smartphones may further exacerbate health disparities for rural patients [38].

Similar to Kayyali et al [20], the majority of the participants were open to data-sharing options with an HCP through mHealth apps. Nevertheless, Kayyali et al [20] stated that data sharing can be ineffective if the participant is not honest or if data sharing is overused.

Some of the findings presented in this study confirm previously reported findings in the context of mHealth for COPD management. Our findings are in agreement with those of Vorrink et al [45], who stressed the importance of training patients and HCPs on the proper use of mHealth. As suggested by Korpershoek et al [15], we confirm that the expected benefits of using mHealth contribute to the success of mHealth uptake, and our study provides additional insight regarding these perceptions, such as on the ease of use, educating patients about mHealth, and the importance of credibility. Our findings echo some and add to the barriers reported by Krebs and Duncan [39], which include technical difficulties and a lack of interest. We included quotes from participants to expand on these insights.

In comparison with the facilitators reported by HCPs, patients had 4 parallel facilitators: there are possible health benefits for patients, the software needs to be easy to use, patients need to be educated on the use of mHealth, and the credibility of mHealth should be evident [29]. The only facilitator that was not mentioned by patients is that mHealth should reduce the cost to the health care system. On the other hand, patients had 5 parallel barriers with the HCPs: there are technical issues with mHealth, lack of awareness is a challenge, there may be limited uptake from the elderly, there are possible financial barriers, and there may be privacy and confidentiality concerns [29]. The possibility of mHealth limiting the personal connection between HCPs and patients was not mentioned as a barrier by patients. Furthermore, one new barrier emerged from interviewing patients; there was little interest in using mHealth interventions. Our findings and the limited literature on this matter emphasize the need for further research into the use of mHealth in COPD management.

### Strengths and Limitations

This study has several strengths. First, we used a mixed methods approach to produce a diverse sample of participants. This human-centered approach ensures that the needs and challenges of a diverse group of patients can be considered before developing an mHealth intervention. Second, some patients had experience in using mHealth interventions to manage their COPD, which further increases the richness of the data. Third, all the interviews were conducted in a similar manner to ensure consistency during data collection and analysis. Finally, we recruited patients with COPD from outpatient respirology clinics. This has led to the capture of a well-characterized cohort of individuals with COPD.

There were also several limitations. First, the number of patients who completed the survey was relatively small. However, all efforts were made to recruit as many participants as possible and facilitate the completion of the survey. Owing to the small sample size, regression coefficients may have been imprecisely estimated. However, the age of the sample was reflective of a representative sample of patients with COPD in Canada who were recruited from a respirology clinic [46]. Furthermore, the main objective of the survey was to obtain a diverse sample of patients with COPD for the qualitative phase. Second, not all patients had experience using mHealth. Thus, the perceptions of these participants were not based on actual interventions with patients. Third, conducting focus groups with some of the participants following the individual interviews could have yielded richer information, as participants would have been given the opportunity to compare their thoughts and confirm or expand upon each other’s ideas. Fourth, there were no questions in the quantitative survey about facilitators of mHealth uptake. Including this topic would have been beneficial, as it could have been expanded upon when conducting the interviews.

### Implications for Practice and Future Research

The findings of this study may help various stakeholders who are planning to use mHealth interventions for COPD management. It is important to consider the low rate of smartphone use among patients when implementing an mHealth intervention for COPD management. Some lessons learned include the importance of raising awareness among patients regarding the potential of mHealth interventions in COPD management. Family members could play a significant role in raising awareness as well as in teaching patients with COPD about mHealth. The findings also emphasize the importance of developing a user-friendly mHealth intervention. This could reduce the time and resources required to teach patients about the mHealth intervention. The lack of an internet connection could limit access to mHealth interventions. This should be taken into consideration when measuring access to health resources in rural communities. Some of the barriers and facilitators have the potential to be applied to other chronic diseases. For example, these findings could be beneficial for stakeholders who plan to develop a mHealth intervention for heart failure or diabetes.

mHealth is particularly important in geographical locations with a relatively large proportion of rural residents such as Newfoundland and Labrador. Of the Atlantic provinces, NL has the highest proportion of its population (60%) living in rural areas [47]. mHealth may enhance care provider access throughout sparsely populated rural areas. Newfoundland and Labrador has a substantial remote and rural population; therefore, our results may be more applicable to rural areas.

Future studies would benefit from conducting focus groups with some of the participants following individual interviews. Focus groups could yield rich information, as participants would be given the opportunity to compare their thoughts and confirm or expand upon each other’s ideas. After developing a user-centered mHealth intervention, the authors recommend using a mixed methods framework for usability testing [48]. Additional trials will be required to provide data regarding the efficacy and cost-effectiveness of mHealth interventions in COPD management.

### Conclusions

It is important to understand access to mHealth among patients with COPD and their perceptions regarding the adoption of mHealth for COPD management. Despite the rise in smartphone adoption, the rate of adoption among patients with COPD remains to be low. Additionally, it is important to consider that owning a smartphone does not mean that one has the ability to use it for mHealth. This finding highlights the need for education and confidence building among some smartphone users to be able to use their devices for COPD management. This study identifies some potential facilitators and barriers that may inform the successful development and implementation of mHealth interventions for COPD management. We recommend that those who develop mHealth interventions for COPD should consider the facilitators and barriers highlighted in this study.

## Appendix

Multimedia Appendix 1 Patient questionnaire.

Multimedia Appendix 2 Patient interview prompts.

## Abbreviations

aOR: adjusted odds ratio
COPD: chronic obstructive pulmonary disease
cOR: crude odds ratio
HCD: human-centered design
HCP: health care professional
ISO: International Organization for Standardization
mHealth: mobile health
