# Supplementary material for: Perceptions of Patients Regarding Mobile Health Interventions for the Management of Chronic Obstructive Pulmonary Disease: Mixed Methods Study
Source: JMIR Mhealth Uhealth. 2020 Jul 23;8(7):e17409. doi: 10.2196/17409 (PMC7413289; doi:10.2196/17409)
Supplement: Multimedia Appendix 1 [file mhealth_v8i7e17409_app1.docx]

## Appendix 1 - Patient questionnaire

### A- Eligibility

**1. Have you been diagnosed by a physician as having Chronic Obstructive Pulmonary Disease (COPD), Emphysema, or Chronic Bronchitis?**

- Yes
- No (If you have not been diagnosed by a physician as having COPD, emphysema, or chronic bronchitis then we’re sorry. You are not eligible to participate in this study. Please discard the questionnaire).

**2. How old are you?**

| - 30- 34 years - 35 - 44 years - 45 - 54 years |  | - 55 - 64 years - Age 65 or older |
| --- | --- | --- |

### B- Mobile Health Technology Ownership

**3. Do you own an iPad and/or tablet?**

- Yes
- No

**4. Do you own a mobile/cell phone?**

- Yes
- No (skip to question 6)

**5. Is the mobile/cell phone you use a smartphone?**

- Yes
- No
- Don’t know

**6. Other than you, does someone in your household have a smartphone?**

| - Yes - Don’t know |  | - No - Live alone |
| --- | --- | --- |

**7. Do you own any of the following health devices? Check all that apply**

| - Spirometer/ Peak flow meter - Glucose meter/ sugar level monitor - Blood pressure monitor - Heart rate monitor |  | - Activity counter (e.g. fitbit or apple watch) - Scale - Thermometer - Insulin pump |
| --- | --- | --- |

- Other, please specify:____________________________________

### C- Mobile Health Technology Use

**8. What language do you use on your phone?**

- English
- French
- Other**,** please specify:____________________________________

**9. Do you use any of the social media accounts listed below? Check all that apply**

| - Facebook - Instagram |  | - Twitter - Snapchat |
| --- | --- | --- |

- I do not use social media accounts (skip to question 11)
- Other, please specify:____________________________________

**10. How often do you use your social media networks?**

- Never
- A few times a month
- A few times a week
- About once a day
- More than once a day

**11. Would you be interested in using social media to share your health experience with other people with similar health issues?**

- Yes
- No

**12. Do you know what a smartphone “app” is?**

- Yes
- No (Skip to question 21)

**13. Do you use apps on your smartphone?**

- Yes
- No (Skip to question 21)
- Don’t know

**14. In the past 12 months, did you use health-related apps on your smartphone?**

- Yes
- No
- Don’t know

**15. Would you be interested in using a smartphone app to improve your health?**

- Yes
- No (skip to question 17)

**16. If you are interested in using a health-related app, how often do you think you would you use it to improve your health?**

- Once a week
- Once a day
- 2-3 times per day
- 4-5 times per day
- 6 or more times per day

**17. What particular health-related app feature do you think would be useful? Check all that apply**

| - Medication information - Inhaler technique education - COPD action plan - General information about disease |  | - Medication Reminders - Nutrition information - Mental wellness techniques |
| --- | --- | --- |

- Other, please specify:____________________________________

**18. Would you be comfortable allowing a family member or friend to access health-related information that you shared in an app?**

- Yes
- No

**19. Would you be comfortable allowing your family doctor or other healthcare professionals to have access to your health information that you shared in an app?**

- Yes
- No

**20. Which of the following concerns do you have about the use of smartphone applications (apps)? Check all that apply**

| - They are not easy to use - They take too much time to use - I do not know if they are effective - Apps use a lot of data |  | - There is an extra fee to use the app - Worried about personal information disclosure - Not recommended by a healthcare provider - None of the above |
| --- | --- | --- |

- Other, please specify:____________________________________

**21. Did you access the Internet from your phone during the past 12 months?**

- Yes
- No
- Don’t know

**22. If you use the internet on your smartphone for health needs, which sites do you use? Check all that apply**

| - Google - WebMD - YouTube |  | - Yahoo/Bing/Other search engines - Mayo clinic/PubMed/NIH - Wikipedia |
| --- | --- | --- |

- Other, please specify:____________________________________

**23. Which of the following concerns do you have about smartphones? Check all that apply**

| - Cost of smartphones - They are not easy to use |  | - Reducing face to face interaction - None of the above |
| --- | --- | --- |

- Other, please specify:____________________________________

### D- Demographics and Health Information

**24. What is your sex?**

- Female
- Male

**25. What is your gender?**

- Female
- Male
- Prefer not to answer
- Other, please specify:____________________________________

**26. What is your Marital Status?**

| - Married - Common Law - Single (never married) |  | - Widowed, Separated, or Divorced - Prefer not to answer |
| --- | --- | --- |

**27. What is your highest level of education?**

| - Less than high school - High School Equivalency (GED) - High School |  | - Bachelor’s degree - Master’s degree - PhD/ MD/ JD |
| --- | --- | --- |

- Prefer not to answer
- Other, please specify:____________________________________

**28. Which of the following best describes where you currently live?**

- Rural area, with a population less than 1,000
- Small population centre, with a population between 1,000 and 29,999
- Medium population centre, with a population between 30,000 and 99,999
- Large urban population centre, with a population of 100,000 or more.

**29. What best describes your employment ?**

| - Employed full time - Employed part time - Self-employed |  | - Retired - Student - Unemployed |
| --- | --- | --- |

**30. What is the range that best describes your gross household income in the last year?**

| - Under $20,000 - $20,000-$39,000 - $40,000-$59,000 - $60,000–79,000 |  | - $80,000–150,000 - Over $150,000 - Don’t know - Prefer not to answer |
| --- | --- | --- |

**31. What medical conditions do you have or have had in the past? Check all that apply**

| - Diabetes - Lung disease - Heart disease - Kidney disease |  | - Cancer - Mental Health Issues - Skeletal/ Muscular disease (e.g. Arthritis) - Prefer not to answer |
| --- | --- | --- |

- Other, please specify:____________________________________

**32. How many medications do you take each day? This include medications you may buy without a prescription**

- None
- 1-2
- 3-4
- 4-6
- More than 6
- Prefer to not answer
- Other, please specify:____________________________________

### E- Future Research

**33. Are you willing to participate in a focus group to help develop an app for COPD management?**

- Yes (Please answer question 36)
- No

**34. Are you willing to participate in an individual interview to help develop an app for COPD management?**

- Yes (Please answer question 36)
- No

**35. Are you willing to be contacted for future research?**

- Yes (Please answer question 36)
- No

**Thank you for participating in this study. Your answers to these questions are important to us, and we really appreciate you taking the time to complete this questionnaire.**

Insert the completed form in the **RED** box located at the clinic

**36. If you answered yes to questions 33, 34 and/ or 35, please provide a phone number and/or email below to allow us to contact you.**

*Note that adding your contact information may present some privacy and confidentiality risks. There is always a small risk that data your answers to the questionnaire may be identified. We will use strict measures to protect your privacy.

Name:

Phone number:

Email:

Best time to call:

Tear this page and insert it the **BLUE** box located at the clinic
