# Supplementary material for: Perceptions of Patients Regarding Mobile Health Interventions for the Management of Chronic Obstructive Pulmonary Disease: Mixed Methods Study
Source: JMIR Mhealth Uhealth. 2020 Jul 23;8(7):e17409. doi: 10.2196/17409 (PMC7413289; doi:10.2196/17409)
Supplement: Multimedia Appendix 2 [file mhealth_v8i7e17409_app2.docx]

## Appendix 2 - Patient Interview prompts

- Date
- Years living with COPD

### General perceptions

- Tell me a little bit about your experience with COPD?
- How do you know if your COPD is getting worse?
- How do you manage it?
- Do you have other chronic diseases besides COPD?
- How do you manage it along with your COPD?
- How many medications do you take?
- How do you remember when to take it?
- Do you own any health devices (e.g., spirometer, blood pressure monitor)?
- How often do you use it?
- Does it connect to your smartphone?
- What type of phone do you have?
  - How did you learn how to use it?
  - Do you live with someone who knows how to use it?
  - Does your friends/ family use a smartphone?
  - Do you know what a smartphone “app” is?
  - Do you use apps on your smartphone?
  - In the past 12 months, did you use health-related apps on your smartphone?
  - Did you access the Internet from your phone during the past 12 months?

### Facilitators

- Do you know what mHealth is? (using a phone to improve your health)
- Have you used mhealth before? (if no, go to barriers).
- Tell me about a situation when you have tried to use mhealth/ telehealth.
  - Why this episode?
  - What did you use it for?
  - What data did you collect?
  - How often?
- What motivated you to use mhealth?
- What elements of the intervention do you think are most important?
- Did you do any preparation before using mHealth to manage COPD?
- What is necessary for you to obtain knowledge/experience and keep up to date about mHealth?

### Barriers

- What is limiting you from using a smartphone?
- What is limiting you from using mHealth?
- What difficulties have you experienced when using mHealth?
- How did you solve it?
- Were there any challenges?
  - (financial, employees, technical)?
- Did you experience changes in the contact/bond with your healthcare provider?

### mHealth in COPD Management

- Do you see a role of mHealth in COPD management?
- How do you feel about apps used in COPD management?
- What features do you want the app to include?
  - Education (how often)
  - Survey (how often)
  - Care plan
  - Compatible medical devices ((e.g. spirometer, pulse oximeter, medication adherence device)
  - Health coach
- What else would you like it to do?
- How often do you think you would you use it to manage your COPD?
- Could you tell me whether you would be interested in using it?
- What about any problems or concerns you can see with this?
- Would you be comfortable allowing a family member or friend to access health-related information that you shared in an app? Why/ why not?
- Would you be comfortable allowing your family doctor or other healthcare professionals to have access to your health information that you shared in an app?Why/ why not?
- How does mHealth affect your current COPD management process?
- How about viewing a large amount of data, e.g. heart rate, spirometry, survey?
- Does your healthcare provider see a role of mHealth in COPD management?
- How does your healthcare provider perceive using telehealth/ mHealth to manage COPD?

### Final questions

- Would you like to add anything?
- Would you like to elaborate on anything I asked?

Thank you for participating in this study, your answers to these questions are very important to us, and we really appreciate you taking the time to complete this interview. Please contact me if you have any questions or would like to discuss this topic further.
